# Whole-volume ADC histogram of the brain as an image biomarker in evaluating disease severity of neonatal hypoxic-ischemic encephalopathy

**DOI:** 10.3389/fneur.2022.918554

**Published:** 2022-08-03

**Authors:** Ruizhu Wang, Yanli Xi, Ming Yang, Meijiao Zhu, Feng Yang, Huafeng Xu

**Affiliations:** Department of Radiology, Children's Hospital of Nanjing Medical University, Nanjing, China

**Keywords:** ADC histogram, neonatal, hypoxic ischemic encephalopathy, neonatal behavioral neurological assessment, biomarker

## Abstract

**Purpose:**

To examine the diagnostic significance of the apparent diffusion coefficient (ADC) histogram in quantifying neonatal hypoxic ischemic encephalopathy (HIE).

**Methods:**

An analysis was conducted on the MRI data of 90 HIE patients, 49 in the moderate-to-severe group, and the other in the mild group. The 3D Slicer software was adopted to delineate the whole brain region as the region of interest, and 22 ADC histogram parameters were obtained. The interobserver consistency of the two radiologists was assessed by the interclass correlation coefficient (ICC). The difference in parameters (ICC > 0.80) between the two groups was compared by performing the independent sample *t*-test or the Mann–Whitney *U* test. In addition, an investigation was conducted on the correlation between parameters and the neonatal behavioral neurological assessment (NBNA) score. The ROC curve was adopted to assess the efficacy of the respective significant parameters. Furthermore, the binary logistic regression was employed to screen out the independent risk factors for determining the severity of HIE.

**Results:**

The ADCmean, ADCmin, ADCmax,10th−70th, 90th percentile of ADC values of the moderate-to-severe group were smaller than those of the mild group, while the group's variance, skewness, kurtosis, heterogeneity, and mode-value were higher than those of the mild group (*P* < 0.05). All the mentioned parameters, the ADCmean, ADCmin, and 10th−70th and 90th percentile of ADC displayed positive correlations with the NBNA score, mode-value and ADCmax displayed no correlations with the NBNA score, the rest showed negative correlations with the NBNA score (*P* < 0.05). The area under the curve (AUC) of variance was the largest (AUC = 0.977; cut-off 972.5, sensitivity 95.1%; specificity 87.8%). According to the logistic regression analysis, skewness, kurtosis, variance, and heterogeneity were independent risk factors for determining the severity of HIE (OR > 1, *P* < 0.05).

**Conclusions:**

The ADC histogram contributes to the HIE diagnosis and is capable of indicating the diffusion information of the brain objectively and quantitatively. It refers to a vital method for assessing the severity of HIE.

## Introduction

Neonatal hypoxic-ischemic encephalopathy (HIE) refers to a brain injury attributed to perinatal asphyxia. In severe cases, it can cause permanent neurological damage to children (e.g., lower verbal IQ, impaired motor and cognitive abilities, and cerebral palsy), thereby imposing a serious burden on the family and society (1, 2). Early hypothermia therapy acts as a vital way to improve prognosis, so the early diagnosis of the disease and the quantification of its relationship with behavior are considered very helpful. Existing magnetic resonance imaging (MRI) technology has been critical to the diagnostic work-up, severity determination, and prognosis of HIE (3, 4), in particular diffusion weighted imaging (DWI). DWI is capable of reflecting the organization structure by observing the movement of water molecules (5), the diffusion coefficients of water are expressed as the apparent diffusion coefficients (ADC), thereby reflecting water diffusion and the development of membranes in neuronal and glial cells. Furthermore, ADC can reflect the difference in the tissue structure in different pathological states (6).

However, existing ADC studies showed common limitations. They all took one or several representative layers of images while drawing the ROI manually. The ADC obtained by the mentioned method failed to reflect the heterogeneity of the whole lesion (7), which might cause heterogeneity to be underestimated. Alternatively, the pattern of injury in HIE is complex and changeable, including brain edema, white matter injury caused by congestion, dilation of deep cerebral veins or cerebral infarction; cortical laminar necrosis, basal ganglia necrosis, brain stem necrosis, cerebellum injury, and so on, especially in moderate-to-severe HIE, gray matter and white matter are both involved, lesion range is wide, so we delineated the whole brain area (removed cerebrospinal fluid) as regions of interest (ROIs), our research is meaningful and innovative. Histogram analysis is a promising imaging analysis technology, which is the first-order feature of radiomics. It distributes each voxel in the region of interest into a histogram, and quantitative parameters describing diffusion heterogeneity can be quickly obtained. Histogram analysis might be easy to understand and apply. Histogram utilized the full potential of the ADC to improve the clinical diagnosis and prognosis by analyzing quantitative factors in brain tissues (8). The histogram analysis can be adopted to quantify the distribution of signal intensity in voxels by complying with the routinely acquired clinical ADC. Lesion heterogeneity can be indicated by histogram features describing the statistical interrelationships between adjacent voxels. The existing method has suggested obvious advantages in tumor grading or prognosis assessment (9, 10), and it is also increasingly employed in numerous non-neoplastic diseases (11–13). However, the use of the ADC histogram in HIE has been rarely reported (14, 15), Cauley KA (14) investigated the use of ADC histographic analysis in the evaluation of the diffusion properties of neonatal brain as a function of gestational age and in the evaluation of diffuse hypoxic-ischemic encephalopathy. Sarioglu FC (15) evaluated the efficacy of the MRI-based texture analysis (TA) of the basal ganglia and thalami to distinguish moderate-to-severe hypoxic-ischemic encephalopathy (HIE) from mild HIE in neonates, they found that the Histogram_entropy log-10 value can be used as an indicator to differentiate between moderate-to-severe to mild HIE.

There were no reports conducted on the relationship between the heterogeneity and the clinical behavior quantification by adopting multiple parameters of the ADC histogram, so a novel method is required. The present study aimed to investigate the value of the whole brain heterogeneity by using the whole-brain ADC histogram to assess the brain change and examine the relationship between heterogeneity and neonatal behavioral neurological assessment (NABA) score, as an attempt to lay a quantitative basis for clinical assessment, provide quantitative findings to indicate different stages in neonates with HIE.

## Materials and methods

### Enrolled patients

In the present cross-sectional study, the data of 90 infants with HIE (46 boys and 44 girls) were confirmed in the neonatology department of Children's Hospital affiliated with Nanjing Medical University from July 2015 to September 2019. To be specific, 49 were in the moderate-to-severe group, and 41 were in the mild group. The inclusion criteria were: (1) the diagnosis of HIE conformed to the criteria for judging HIE revised by the neonatology group of pediatrics society of the Chinese medical association in 2005. (2) the mean neonatal gestational age ≥ 38 weeks; (3) the grouping criteria of the mild group and moderate-to-severe group complied with the conventional MRI scan sequence, as reported by reference (16). The exclusion criteria are listed here: (1) congenital diseases and genetic metabolic diseases were excluded. (2) neonatal bilirubin encephalopathy was excluded. (3) those neonates whose poor image quality did not satisfy the diagnostic requirements were excluded. The patients we selected were all term infants whose mean gestational age was ≥ 38 weeks, we ruled out premature infants. Meanwhile, at the age when they did the MRI scanning was 1–9 d, there was no significant difference in neonatal white matter myelination over several days. We tried to minimize the possibility of heterogeneity caused by differentiation levels among patients.

### Imaging protocol

Based on the SIEMENS AVANTO 1.5T superconducting magnetic resonance scanner, with a 32 channel head coil, the patients were given oral 5% chloral hydrate 1 ml/kg prior to the examination, and they were scanned after sleep. The age when they did the MRI scanning was 1–9 d. The sequence and imaging parameters are presented below: the conventional sequence, matrix 256 × 144, the field of vision (FOV) 200 × 200 mm, the layer thickness 5 mm, the layer spacing 2 mm; the axis T1-weighted image (T1WI) spin echo sequence, the repeat time (TR) 400 ms, the echo time (TE) 7.8 ms; the fast spin echo sequence with T2WI, TR 3,250 ms, TE 99 ms; the axial T2 liquid attenuating inversion recovery sequence (FLAIR), TR 9,000 ms, TE109 ms; the sagittal fast spin echo sequence, TR 400 ms, TE 7.8 ms; the axial dispersion weighted image (DWI) sequence, TR 400 ms, TE 7.8 ms, *B* value 0 s/mm^2^, 750 s/mm^2^, matrix 130 × 130, visual field (FOV) 220 × 200 mm, layer thickness 5 mm, layer spacing 2 mm. The head MRI scan analysis was conducted by two radiologists (with 8 and 9 years of experience in the interpretation of neurology MRI images), respectively, and the results were agreed upon after the discussion.

### Image acquisition and feature extraction

The ADC images were exported from a PACS workstation in DICOM format, and then they were generated with a monoexponential fit of diffusion data from the *b*-values of 0 and 750 s/mm^2^ using the following formula: ADC = [lnS0–lnS(*b*)]/*b* (where *S*0 and *S*(*b*) represent the DWI signal intensity at *b* = 0 and 750 s/mm^2^, respectively). An open-source software 3D Slicer (https://www.slicer.org/), was used to extract all the histogram features (17, 18). Two independent observers (with 8 and 9 years of experience in the interpretation of neurology MRI images), blinded to the pathologic and biochemical findings, manually delineated the whole brain area (removed cerebrospinal fluid) as regions of interest (ROIs) in the respective slice on the ADC diagrams to encompass the whole brain volume. Then whole-brain ADC histograms were obtained. Histogram analysis describes the statistical information. In this study, the ADCmean value (ADCmean), the ADC minimum value (ADCmin), the ADC maximum value (ADCmax), and the 10th−90th percentile of ADC value [The *N*th percentage represents the point found from the left that constitutes the *N*th voxel value of the histogram (19)], the skewness, the kurtosis, the entropy, the mode-count, the mode-value, the variance, the contrast, the heterogeneity, the homogeneity, and the angular second moment (ASM) were calculated, there were 23 histogram parameters in all. The absolute correlation coefficient (ACC) between the features with Spearmen's rank correlation was calculated to remove highly correlated features.

### Determine the NBNA score of neonates

According to the 20 scoring criteria formulated by Professor Bao Xiulan of Peking Union Medical College Hospital (20), all neonates with HIE were scored within 9 days after birth by trained neonatal physicians. In the state of quiet sleep, the examination was conducted on 6 items of behavioral ability, 4 items of active muscle tone, 4 items of passive muscle tone, 3 items of original reflex, and 3 items of general response. Each item is scored on three scales, i.e., 0, 1, and 2, for a total of 40 points. A score ≥35 was considered normal, while a score < 35 was considered abnormal.

### Statistics

All data were analyzed with SPSS20.0. A Kruskal–Wallis test was performed to compare the genders. The Chi-squared test was performed to compare gestational age and age between the two groups. The interobserver consistency of the measurements between the two radiologists was assessed by determining the interclass correlation coefficient (ICC). Parameters with an ICC <0.8 were excluded from further statistical analyses. Kolmogorov–Smirnova was used to determine whether the variables had a normal distribution. An independent sample *t*-test or Mann–Whitney *U* test was performed to compare the difference between the two groups, *P* < 0.05 was considered statistically significant. The Pearson correlation analysis was performed with statistically significant parameters and the NBNA value, *P* < 0.05 was statistically significant. The receiver operating characteristic (ROC) curve was adopted to determine the potential diagnostic performance for differentiating the severity of HIE. The optimal threshold was selected according to the Youden index. The statistically significant parameters were taken as the independent variables, and the severity of HIE was taken as the dependent variable. Furthermore, the binary logistic regression analysis was conducted, and *P* < 0.05 was statistically significant.

## Results

### Demographic data

A total of 49 cases were recruited in the moderate-to-severe group, and 41 cases were enrolled in the mild group. The gestational age of the moderate-to-severe group: age was 39.1 ± 0.5 (38.2~40.3) weeks, the age at scanning was 5.9 ± 2.8 (3~10) d, the weight was 3190 ± 190 (3010–3970) g. The gestational age of the mild group was 39.4 ± 0.3 (38.1–40) weeks, the age at scanning was 6.8 ± 1.4 (4–12) days, and the weight was 3230 ± 240 (3130–3890) g. In the moderate-to-severe group, two severe ill patients died due to the abandonment of treatment after the MRI examination. Furthermore, the age and sex of the two groups were matched. No significant difference was reported in gender, age, weight, gestational age, and the age at scanning between the two groups (*P* > 0.05) (Table 1).

**Table 1:** Demographic variables of enrolled patients.

	**Gender (male:female)**	**Age** **(days)**	**Gestational age (weeks)**	**Weight (g)**	**Age at scanning (weeks)**
mild group	22:19	10.0 ± 3.4	39.4 ± 0.3	3230 ± 240	6.8 ± 1.4
moderate-to-severe group	24:25	9.0 ± 3.2	39.1 ± 0.5	3190 ± 190	5.9 ± 2.8
*P*	0.890	0.210	0.090	0.152	0.103

### Results of the NBNA value

**Figure 1:**
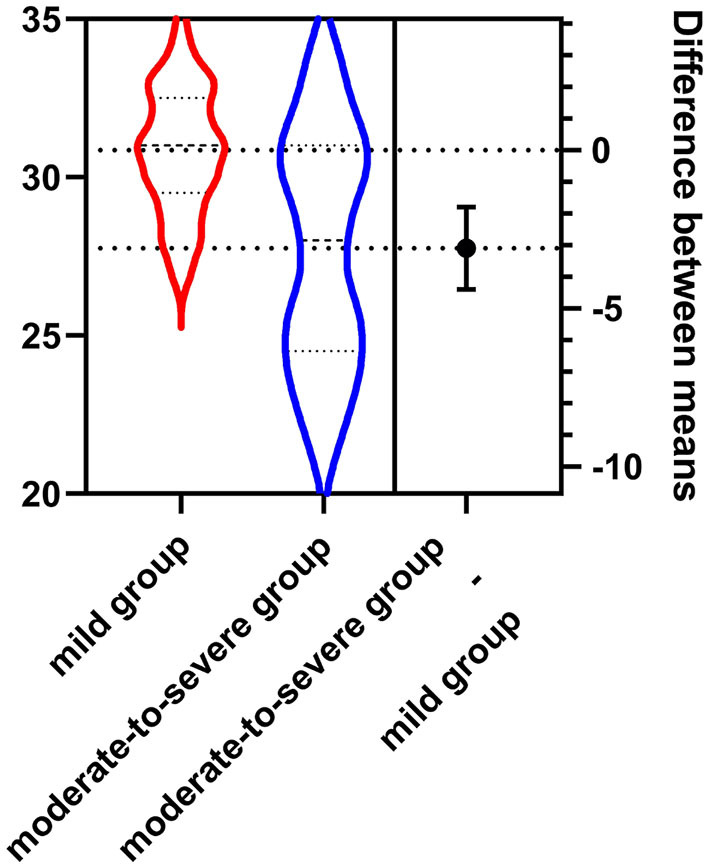
The NBNA score of the mild group (30.85 ± 1.62) was higher than that of the moderate-to-severe group (27.76 ± 2.24).

The NBNA score of the mild group was higher than those of the moderate-to-severe group, *P* < 0.001(as shown in Table 2, Figure 1).

**Table 2:** Comparison of the NBNA value between two groups.

	**Cohort**	**NBNA score**
mild group	41	30.85 ± 1.62
moderate-to-severe group	49	27.76 ± 2.24
*t*		2.13
*P*		0.000

*NBNA, neonatal behavioral neurological assessment*.

### Interobserver agreement

For all the parameters except for the mode-count and 80th percentile of ADC (ICCs <0.8), the interobserver agreement between two observers was prominent (ICCs ranging from 0.912 to 0.976). Thus, the mode-count and 80th percentile of ADC value were excluded. The other parameters were further assessed.

### Results of ADC histogram parameters in two groups

**Figure 2:**
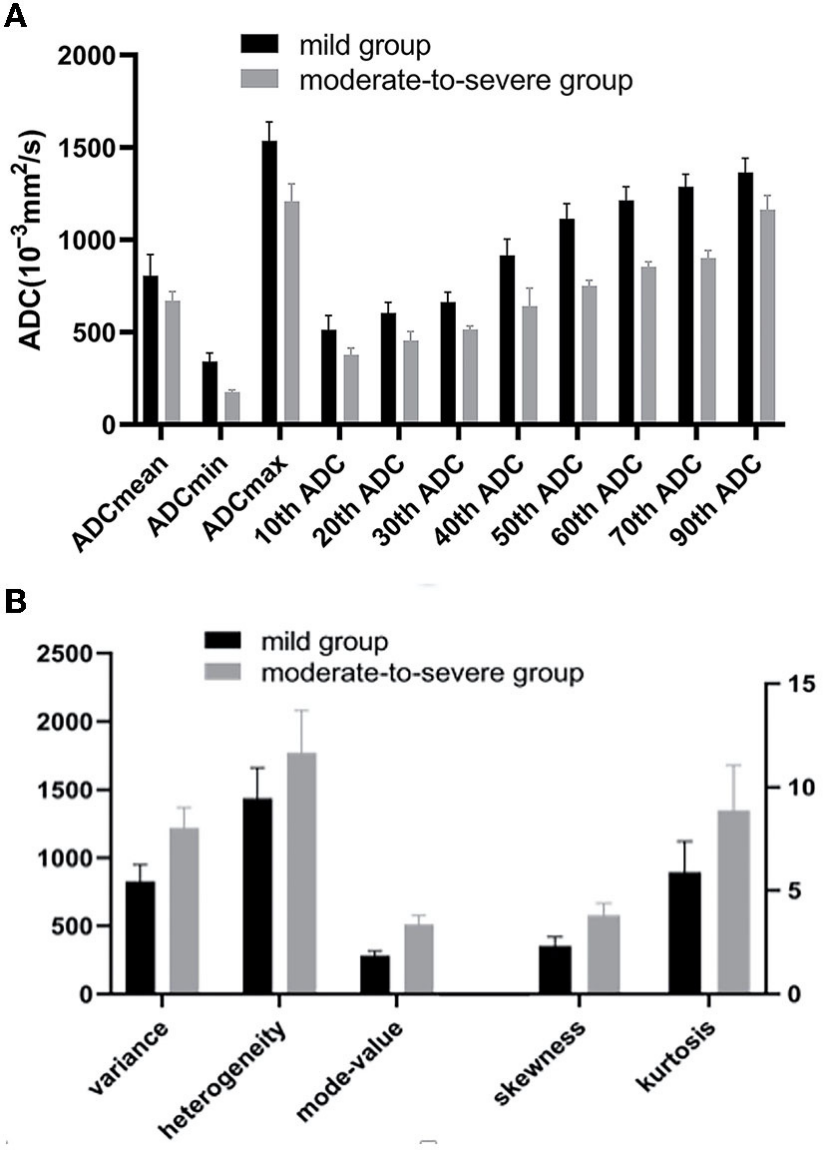
**(A)** The ADCmean, ADCmin, ADCmax,10th-70th and 90th percentile of ADC values of the moderate-to-severe group were all lower than that of the mild group (*P* < 0.05). **(B)** The Variance, the skewness, the kurtosis, the heterogeneity, and the mode-value of the moderate-to-severe group were larger than that of the mild group (*P* < 0.05). ADC, apparent diffusion coefficients (10^−3^mm^2^/s).

**Figure 3:**
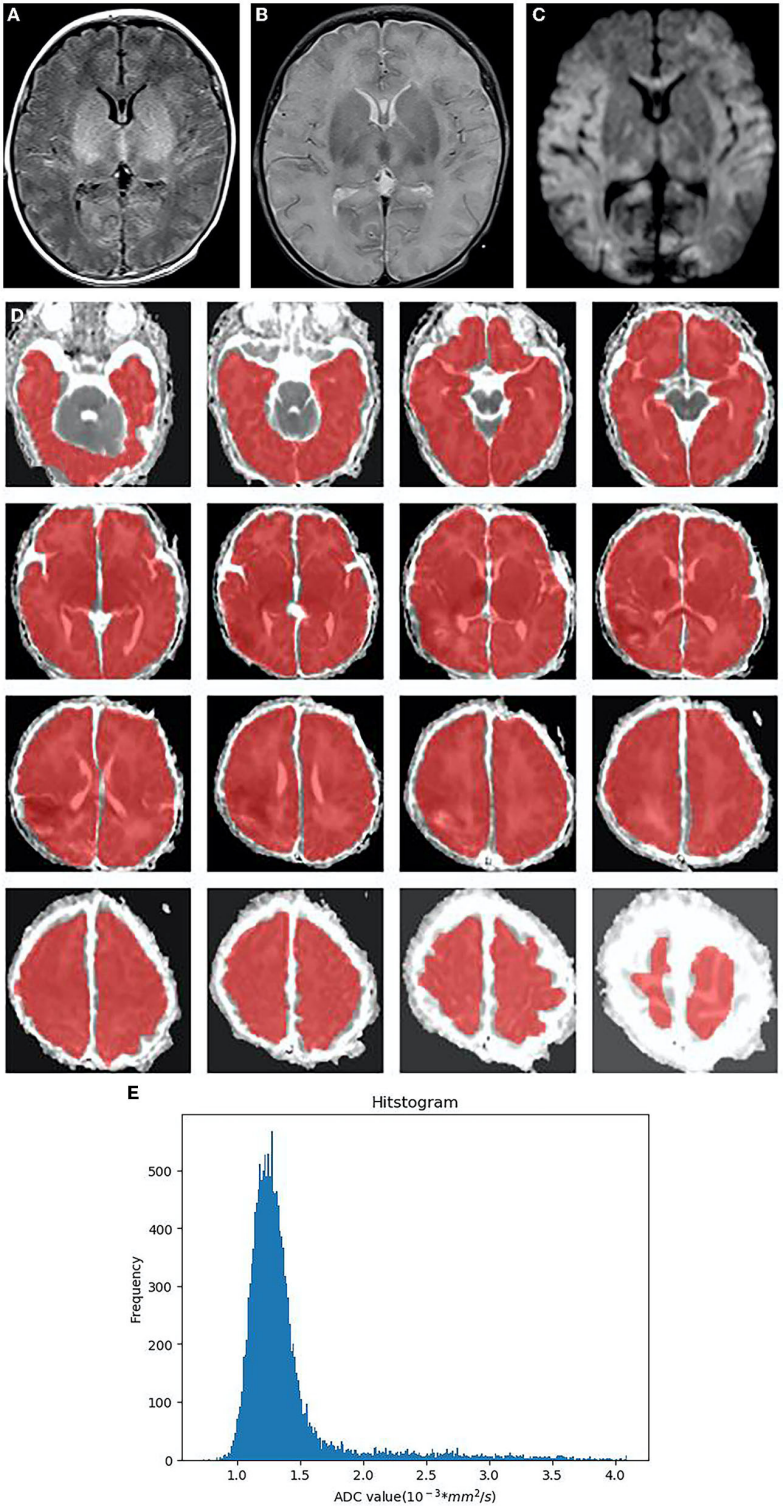
Neonatal with HIE, moderate-to-severe group, male, 4 days old. **(A)**:T1WI sequence, the blurred boundary between gray and white matter in bilateral frontal, temporal lobes, and occipital lobes, the decreased signal in white matter, the increased signal in the thalamus and basal ganglia, as well as the disappeared signal in the posterior limb of the internal capsule. **(B)** T2WI sequence, brain swelling, the increased signal in white matter, the decreased signal in the thalamus and basal ganglia. **(C)** DWI, the extensive high signal in the frontal lobe, temporal lobe, occipital lobe, thalamus and corpus callosum on DWI **(D)** In ADC diagram, the whole brain area (CSF removal) was manually delineated as the regions of interest (ROIs) in each slice. **(E)** The histogram of ADC shows the positive skew distribution of asymmetric frequency distribution. The *X*-axis represents the ADC value, and the *Y*-axis denotes the pixel frequency corresponding to the ADC value. HIE, neonatal hypoxic-ischemic encephalopathy; CSF, cerebrospinal fluid; ROI, regions of interest.

**Figure 4:**
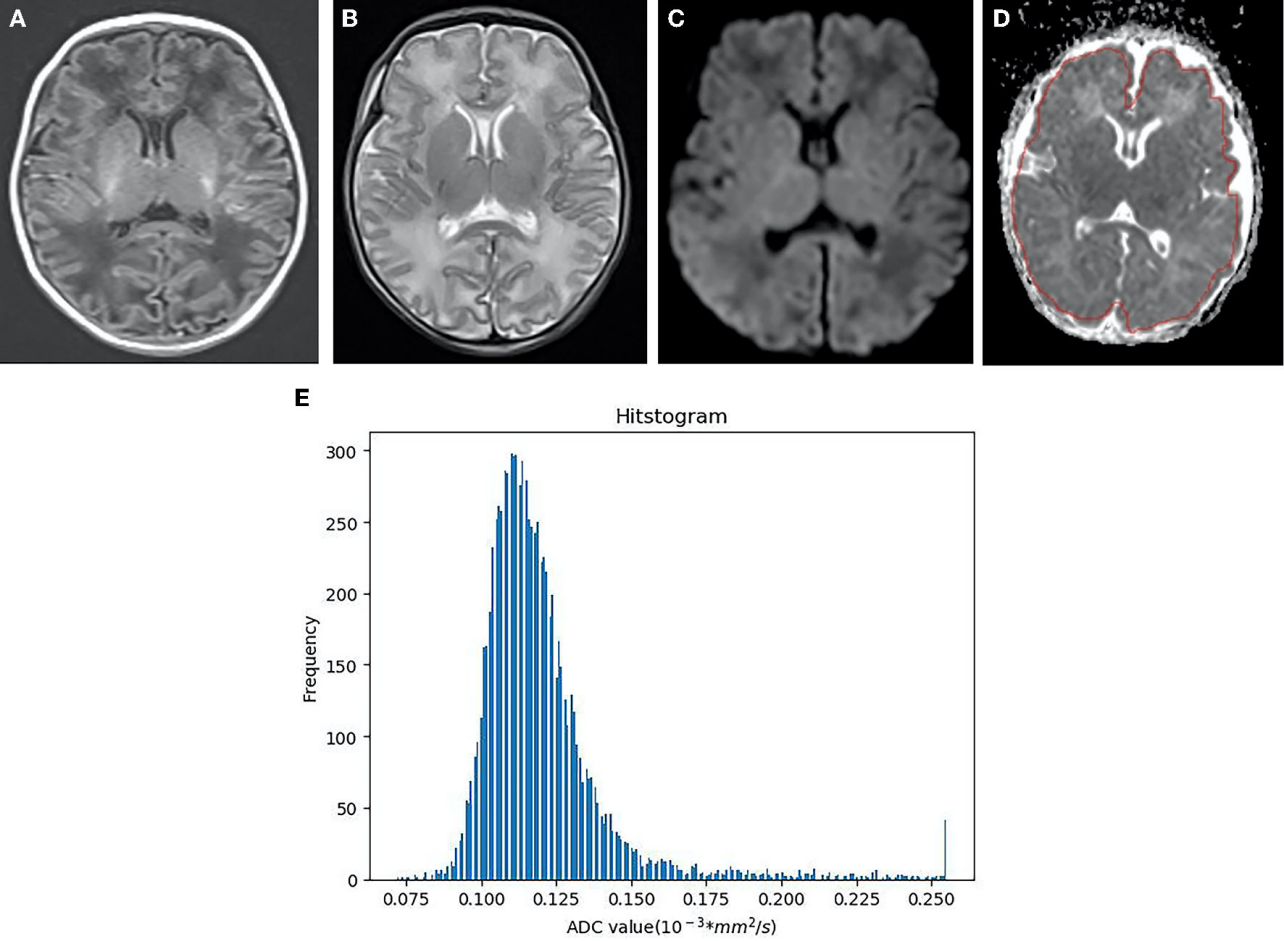
Neonatal with HIE in the mild group, female, 6 days old. **(A)** T1WI sequence, punctate and striped T1 high signal in bilateral frontal parietal cortex and subcortical cortex. **(B)** T2WI sequence, the white matter signal is slightly higher. **(C)** DWI, punctuate hyper signal close to the left lateral ventricle. **(D)** The identical area was selected as that in the moderate-to-severe group to draw ROI. **(E)** As indicated from the ADC histogram of the mild group, the distribution of the ADC values is more symmetrical than that of the moderate-to-severe group, the distribution width is narrower, and the highest frequency value is lower than that of the moderate-to-severe group (*X*-axis represents ADC value, *Y*-axis denotes the pixel frequency corresponding to ADC value).

The absolute correlation coefficient between mode-count and angular second moment (ASM) was 0.897, the absolute correlation coefficient between contrast and homogeneity was 0.912, and the four features were eliminated. The ADCmean, ADCmin, ADCmax, 10th−70th and 90th percentile of ADC value of the moderate-to-severe group were all lower than that of the mild group. The variance, the skewness, the kurtosis, the heterogeneity, and the mode-value of the moderate-to-severe group were larger than that of the mild group, and the difference was statistically significant (*P* < 0.05) (Figures 2–4).

### Results of correlation analysis

The Pearson correlation analysis showed that the ADCmean, ADCmin, 10th−70th, and 90th percentile of ADC values of the HIE displayed positive correlations with the NBNA value. The variance, the kurtosis, the heterogeneity, and the skewness were negatively correlated with the NBNA value (*P* < 0.05). The mode-value and the ADCmax had no correlation with the NBNA vs. value (*P* > 0.05) (as shown in Table 3).

**Table 3:** Correlation between parameters and the NBNA value.

**Parameter**	**NBNA score**	
	***r***	***P***
ADC mean	0.768	0.001
ADC max	0.312	0.089
ADC min	0.755	0.001
10th ADC	0.883	0.000
20th ADC	0.684	0.000
30th ADC	0.582	0.000
40th ADC	0.678	0.000
50th ADC	0.574	0.000
60th ADC	0.471	0.001
70th ADC	0.567	0.001
90th ADC	0.656	0.001
variance	−0.334	0.019
kurtosis	−0.612	0.029
skewness	−0.668	0.003
heterogeneity	−0.508	0.000
mode-value	−0.122	0.168

### Diagnostic performance

**Figure 5:**
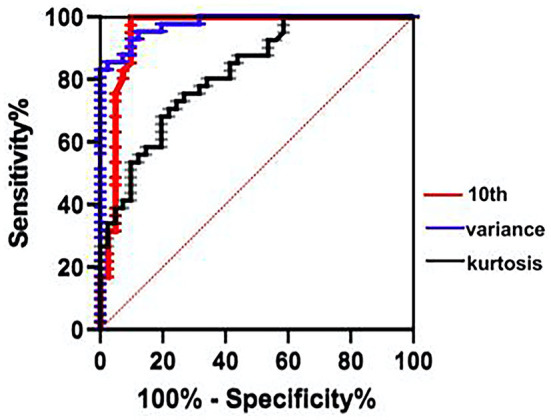
ROC curve indicated that the variance has the greatest diagnostic efficacy for the moderate-to-severe HIE (cut-off = 972.5, sensitivity 95.12%, specificity 87.8%), followed by the 10th percentile of ADC value (AUC = 0.954), the kurtosis (AUC = 0.878). ROC, receiver operating characteristic; AUC, area under the curve.

The ROC curve indicated the efficacy of ADC histogram parameters in the diagnosis of moderate-to-severe HIE, the area under the curve (AUC) of variance was the highest (0.977). Under the cut-off value of 972.5, the sensitivity and specificity were 95.1% and 87.8%. Diagnostic efficacy was followed by the 10th percentile of ADC value (AUC = 0.954) and the kurtosis (AUC = 0.878) (Figure 5).

### Logistic regression analysis

**Figure 6:**
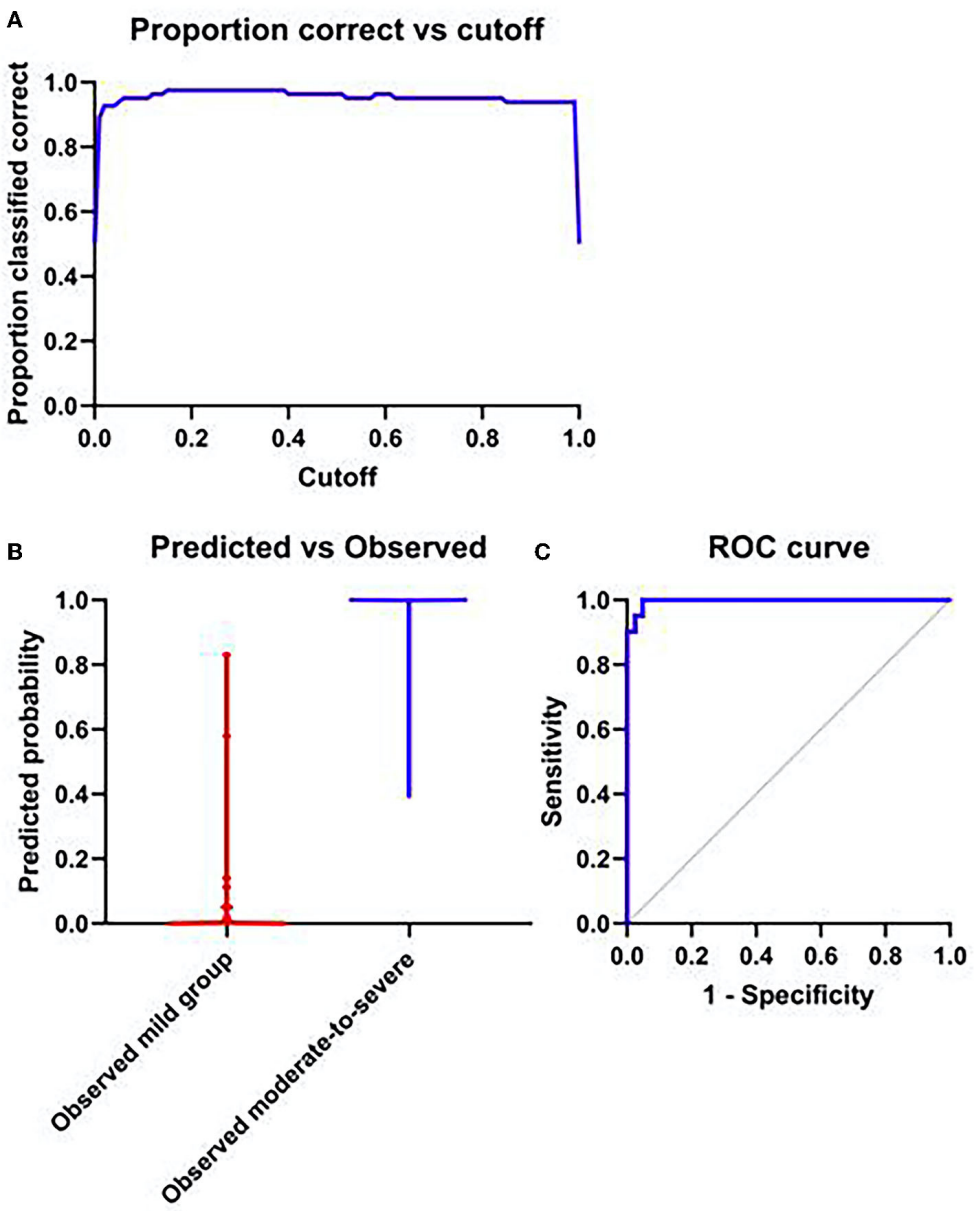
**(A)** The probability of accurate prediction changes with different prediction thresholds. **(B)** Forecast probability and measured individual. **(C)** ROC curve of model; AUC = 0.996.

The skewness (OR = 25.84, 95%CI:3.13–160.24, *P* = 0.001), the kurtosis (OR = 4.122, 95%CI: 1.066–11.233, *P* = 0.003), the variance (OR = 1.047, 95%CI:1.011–1.321, P = 0.02) and the heterogeneity (OR = 1.018, 95%CI:1.0–1.196, *P* = 0.03) were independent risk factors for the diagnosis of moderate to severe HIE (*P* < 0.05), ROC curve of the model (AUC = 0.996), shown in Figure 6.

## Discussion

The histogram analysis complies with the voxel-wise apparent diffusion coefficient (ADC) value distribution, which has been increasingly conducted. Over the past few years, the detection of tumor spatial heterogeneity by employing medical images has aroused considerable attention for its non-invasive nature and overall assessment of the whole tumors compared with biopsy. Numerous mathematical methods have been developed and employed to characterize the tumor heterogeneity, of which the whole-lesion histogram analysis acts as a highly available and popular method (21). With the development of post-processing platforms, the whole-lesion histogram analysis has been facilitated, thereby making it easy-to-use and less time-consuming. In this study, the whole brain region was delineated, and the data were analyzed. The ADC value and the pixel value in the identical range acted as the *X*-axis and *Y*-axis, respectively, to generate the histogram of the ADC, and the average signal value of the whole brain region, pixel value, and pixel variation range were measured for the histogram analysis.

The ADCmean in the histogram is capable of reflecting the central trend and average level of the data, and the ADCmin can reflect the ADC value of the most severe injury in the brain of HIE. As reported by Kang (22), low percentile ADC values were significantly correlated with high cell density. In this study, the ADCmean, ADCmin, and the 10th−70th and 90th percentile of ADC values in the moderate-to-severe group were lower than those in the mild group, which demonstrated that the limited diffusion of water molecules in the brain of the moderate-to-severe group reached over that of the mild group, which explained the edema of brain cell attributed to energy metabolism disorders after HIE. The variance and heterogeneity are recognized as two vital parameters to describe the heterogeneity of lesions (23, 24). The variance and heterogeneity of the moderate-to-severe group exceeded those of the mild group, which demonstrated that the heterogeneity of the ROI was large and that the complexity of the image was high, which could objectively reflect the pathological variations of brain edema, degeneration, necrosis, and other pathological changes after hypoxia, and could accurately identify the lesions identifiable by morphology. Entropy represents the statistical measure of variation that can be exploited to characterize the image texture. Entropy increases as the data distribution become more irregular. No statistical difference was reported in entropy between the two groups, and its significance needs to be confirmed in depth.

The kurtosis of the histogram can describe the degree of steepness and gentleness of the overall data distribution in the lesion. With the normal distribution as a reference, positive kurtosis indicates that the data distribution is sharper than the normal distribution, while negative kurtosis reveals that the data distribution is flatter. Skewness in the histogram indicates the degree of uneven histogram distribution. When the skewness value is negative, the “tail” on the left is longer than that on the right, which is termed negative skewness distribution. The ADC values of most voxels in the ROI are suggested to be concentrated in the higher part; as opposed to the mentioned, if the skewness value is positive, it is termed as positive skewness distribution. The ADC values representing most voxels in the ROI are concentrated in the lower part. In existing studies, differences in kurtosis and skewness varied, Ma Gao (25) studied the benign and malignant tumors in the head and neck and reported no significant difference in kurtosis and skewness. In the case of non-neoplastic lesions, Zhu Meijiao (26) examined the brain tissue of neonates with congenital heart disease with the ADC histogram, and she found that the kurtosis and skewness of the brain of children with congenital heart disease exceeded those of the control group. In this study, the kurtosis of the moderate-to-severe group was higher than that of the mild group, possibly because of the degeneration and necrosis of nerve cells, the increase in the vascular permeability, and the proliferation of glial cells, thereby causing the diffusion of water molecules away from the normal distribution. The skewness of the moderate-to-severe group was higher, displaying a positive skewness distribution, which might be attributed to more serious limited brain tissue diffusion attributed to insufficient hypoxic and ischemic energy supply, brain edema, cerebral hemorrhage, etc., resulting in the concentration of most of the voxel ADC values in the lower part.

Correlation analysis found that all parameters with statistical significance displayed correlations with the NBNA value except the mode-value and the ADCmax, which were not correlated with the NBNA value. The NBNA value is a scoring method established by Chinese scholars based on the Brazelton Neonatal Behavior Score from the United States and the Amiel-Tison Neuromotor Measurement Method from France. It refers to a comprehensive neurobehavioral examination method, capable of comprehensively assessing the neonatal behavioral ability, various nerve reflexes, and states. Besides, it is more accurate and perfect than the conventional neurological examination. Mild brain injury can be detected early. The NBNA value is easy to operate and has high sensitivity and specificity for early detection of brain injury, which can be used as a reliable indicator for early assessment (27, 28) and has been widely used in clinical practice. In this study, it was found that most of the parameters with statistical significance displayed correlations with the NBNA value, since the lower the NBNA score, the more severe the brain injury was. Accordingly, the mentioned ADC histogram parameters could effectively predict and assess the neonatal brain injury and judge the severity of the disease, which could provide more quantitative information for clinicians. The ROC curve showed the highest area under the curve (AUC) of variance (AUC = 0.977). Under the cut-off value of 972.5, the sensitivity and specificity were 95.1% and 87.8%, followed by the 10th percentile of ADC(AUC = 0.954) and kurtosis (AUC = 0.878). Binary logistic regression analysis screened four statistically significant variables, i.e., the Skewness, the kurtosis, the variance, and the heterogeneity (OR > 1, *P* < 0.05), which demonstrated that compared with other parameters, they were independent risk factors for the diagnosis of moderate to severe hypoxic ischemic encephalopathy. OR of skewness is the largest (25.84, 95% CI: 3.13–160.24) indicating that skewness had the greatest diagnostic significance for identifying moderate to severe HIE. The mentioned four parameters could be observed to identify HIE in combination with other characteristics.

In brief, compared with the conventional ROI-based method, the ADC histogram method adopted in this study exhibited higher reproducibility, more diagnostic parameters, and higher diagnostic efficiency. ADC histogram might be a potential biomarker of alterations in tissue structure, thereby laying an objective basis for clinicians to diagnose. Despite the great potential, considerable challenges remain; for an instant, the standardization, reproducibility, and biological validation should be addressed before they can play a role in clinical decision-making. More follow-ups should be conducted to verify the accuracy of the study.

The defects of this study are as follows: (1) The ADC histogram of monolayer brain tissue did not cover all the voxels of the whole brain, which might cause sampling bias. (2) The signal-to-noise ratio (SNR) of the ADC diagram was poor. When the ROI was delineated, the ventricle, sulci, and cistern of the brain could not be overall removed, which certainly impacted the data measured. (3) In addition, only a single *b*-value was selected in this study, which showed certain limitations. It is known that the conventional ADC value derived from the monoexponential model was impacted by a combination of both diffusion and perfusion effects. Intra-voxel incoherent motion (IVIM) imaging may tease out the respective component from the total DWI signal, whereas IVIM imaging requires a series of *b*-values (at least three) (29). Some scholars compared the multi-*b* value ADC histogram and suggested the different diagnostic efficiency of glioma (22). Subsequent studies should be conducted to determine whether the multi-b value ADC histogram is different in HIE (4) The examination time of the majority of cases was selected in 7 d after birth, whereas some severe HIE newborns could not get off the ventilator and warm box at the early stage. For this reason, the examination time was later, which might have led to the lesions in the ADC figure during the pseudo-normalization (30), which would interfere with the results. Subsequent studies should improve the deficiencies and explore the neuropathological mechanism of HIE, in an attempt to provide evidence for its early clinical diagnosis and treatment.

## Data availability statement

The original contributions presented in the study are included in the article/supplementary material, further inquiries can be directed to the corresponding author.

## Ethics statement

The studies involving human participants were reviewed and approved by Medical Ethics Committee, Children's Hospital affiliated to Nanjing Medical University. Written informed consent to participate in this study was provided by the participants' legal guardian/next of kin. Written informed consent was obtained from the individual(s) and minor(s)' legal guardian/next of kin, for the publication of any potentially identifiable images or data included in this article.

## Author contributions

YX and HX prepared the data and drafted the medical part of the manuscript. RW analyzed the data and drafted the manuscript. FY and MZ extracted data features and investigated the patients' clinical data. MY guided and revised the manuscript throughout. All authors contributed to manuscript development, and read and approved the final manuscript.

## Funding

Six Talent Peaks Project in Jiangsu Province, CN (WSN-192); China and Jiangsu commission of health, CN (LGY2019009); Nanjing Science and Technology Development Fund, CN (YKK14117).

## Conflict of interest

The authors declare that the research was conducted in the absence of any commercial or financial relationships that could be construed as a potential conflict of interest.

## Publisher's note

All claims expressed in this article are solely those of the authors and do not necessarily represent those of their affiliated organizations, or those of the publisher, the editors and the reviewers. Any product that may be evaluated in this article, or claim that may be made by its manufacturer, is not guaranteed or endorsed by the publisher.
